# *N*-acetylcysteine exposure is associated with improved survival in anti-nuclear antibody seropositive patients with usual interstitial pneumonia

**DOI:** 10.1186/s12890-018-0599-3

**Published:** 2018-02-08

**Authors:** Justin M. Oldham, Leah J. Witt, Ayodeji Adegunsoye, Jonathan H. Chung, Cathryn Lee, Scully Hsu, Lena W. Chen, Aliya Husain, Steven Montner, Rekha Vij, Mary E. Strek, Imre Noth

**Affiliations:** 1 Department of Internal Medicine, Division of Pulmonary, Critical Care and Sleep Medicine, The University of California at Davis, Sacramento, CA USA; 2 Department of Medicine; Division of Geriatrics, University of California at San Francisco, San Francisco, USA; 3 Department of Medicine; Section of Pulmonary and Critical Care Medicine, The University of Chicago, Chicago, USA; 4 Department of Radiology, The University of Chicago, Chicago, USA; 5 Department of Pathology, The University of Chicago, Chicago, USA

**Keywords:** Idiopathic pulmonary fibrosis, Interstitial lung disease, Interstitial pneumonia with autoimmune features, Anti-nuclear autoantibody

## Abstract

**Background:**

Mortality is similarly high among individuals with usual interstitial pneumonia (UIP) due to idiopathic pulmonary fibrosis (IPF) and interstitial pneumonia with autoimmune features (IPAF). Circulating anti-nuclear antibodies (ANA) are commonly found in this patient population, suggesting possible aberrant immune activation. Because an environment of oxidative stress can result from immunologic activation, we hypothesized that ANA positive patients with UIP would have improved outcome when exposed to the antioxidant *N*-acetylcysteine (NAC) compared to ANA negative patients.

**Methods:**

A single center, retrospective cohort analysis was performed. Patients with UIP due to IPF and IPAF were stratified according to ANA status to and NAC exposure. Transplant-free survival (TFS) was assessed using the Kaplan-Meier estimator and multivariable Cox regression adjusted for diagnosis, gender/age/physiology score, immunosuppressant exposure and anti-fibrotic exposure.

**Results:**

Of 293 individuals with UIP due to IPF (74%) or IPAF (26%), NAC exposure was documented in 58 (19.8%). Among NAC exposed individuals, 33 (56.9%) were ANA seropositive and 25 (43.1%) were seronegative. NAC exposure was associated with improved TFS survival among ANA seropositive individuals in unadjusted analysis (p_logrank_ = 0.02) and after multi-variable adjustment (HR 0.51, 95% CI 0.30–0.87; *p* = 0.01). There was no association between NAC exposure and TFS in ANA seronegative individuals (HR 1.26, 95% CI 0.69–2.32; *p* = 0.45). Formal interaction testing confirmed NAC*ANA interaction (*p* = 0.04) and sensitivity analysis demonstrated an increasing effect size associated with NAC therapy as ANA titer increased. Among patients with available genetic data, a marginally higher proportion of ANA positive patients (*p* = 0.08) carried the rs3750920 (*TOLLIP*) genotype previously shown to predict favorable outcome in NAC exposed patients.

**Conclusion:**

NAC exposure is associated with improved transplant-free survival ANA positive patients with UIP. These findings support the prospective collection of ANA data in in future NAC clinical trials performed in patients with UIP.

**Electronic supplementary material:**

The online version of this article (10.1186/s12890-018-0599-3) contains supplementary material, which is available to authorized users.

## Background

Idiopathic pulmonary fibrosis (IPF) is a fibroproliferative interstitial lung disease (ILD) of unknown etiology that results in a progressive loss of lung function and median survival of 3–5 years [1–3]. Some individuals with IPF and other forms of idiopathic interstitial pneumonia (IIP) display features of connective tissue disease (CTD), but fail to meet established criteria for a specific CTD. Recognition that such individuals may represent a unique phenotype led to a recent American Thoracic Society (ATS)/European Respiratory Society (ERS) joint research statement that proposed criteria for interstitial pneumonia with autoimmune features (IPAF) [4]. After applying IPAF criteria to patients with IPF and other ILDs, we and other investigators have shown that IPAF survival was similar to that of IPF, [5, 6] especially among those with usual interstitial pneumonia (UIP) [6].

While the optimal therapy for patients with IPAF has yet to be established, a host of randomized clinical trials have been performed in patients with IPF, including several that demonstrated the efficacy of the anti-fibrotic compounds pirfenidone and nintedanib in slowing pulmonary function decline [7–9]. Prior to the approval of anti-fibrotic therapy for IPF, immunosuppressant and anti-oxidant therapies were routinely used. This practice effectively ended following the PANTHER trial, due to increased mortality in those treated with a combination of prednisone, azathioprine and *N*-acetylcysteine (NAC) and lack of efficacy in those treated with NAC monotherapy [10, 11].

Our group recently showed that treatment with NAC therapy may improve outcomes among individuals with IPF who carry an rs3750920 TT genotype in *TOLLIP* [12]. *TOLLIP* encodes toll-interacting protein, which regulates downstream inflammatory signaling by interaction with toll-like receptors (TLRs) [13, 14]. The TLRs are critical mediators of airway and alveolar host defense through recognition of microbial antigens [15]. TLR activation can also occur upon recognition of self-antigens bound to autoantibodies, leading to systemic inflammation characteristic of systemic autoimmune disease [16–19]. A commonly encountered circulating autoantibody is the anti-nuclear antibody (ANA), which is checked in all patients undergoing ILD evaluation at our institution, and has been described in up to 35% of patients with IPF [20] and 82% of patients with IPAF [5]. Because antioxidants have been shown to mitigate experimental models of ANA production [21] and modulate oxidative stress caused by TLR activation, [22–24] we hypothesized that NAC responsiveness would vary based on ANA status. To test this hypothesis we conducted a retrospective, ANA-stratified cohort analysis of survival in NAC exposed vs. non-exposed individuals with UIP due to IPF and IPAF.

## Methods

### Study design

This investigation was conducted at the University of Chicago and was approved by the University of Chicago Institutional Review Board (protocol #14163-A). All patients included in this study provided written informed consent. The University of Chicago ILD registry was used to identify consecutive patients followed from October 2006 to January 2016 with a multi-disciplinary diagnosis of IPF or IPAF according to ATS/ERS consensus guidelines [4, 25]. The electronic medical record was retrospectively reviewed to extract pertinent data. A patient was considered to have therapy exposure when he or she 1) endorsed ongoing use of a medication at the time of first ILD evaluation or 2) was prescribed at least 3 months of a medication after establishing care at our institution. ANA seropositivity was defined as an ANA titer ≥1:320 or nucleolar or centromere staining pattern at any titer, in accordance with proposed IPAF criteria [4].

Other information gathered included demographic information (age, gender, race/ethnicity), patient-reported medication use including NAC, azathioprine, mycophenolate mofetil, prednisone, pirfenidone and nintedanib, laboratory studies including antinuclear antibody (ANA) with immunofluorescence pattern and other autoantibodies (rheumatoid factor, anti-cyclic citrullinated protein and anti-SSA, anti-SSB, anti-RNP, anti-Smith and anti-Scl-70 antibodies) and pulmonary function tests, including percent predicted forced vital capacity (FVC), and percent predicted diffusion capacity of the lung for carbon monoxide (D_LCO_).

High-resolution computed tomography (HRCT) scans were reviewed by two chest radiologists with ILD expertise (JC and SM) to determine a consensus radiographic pattern. IPAF criteria were applied to HRCTs and surgical lung biopsies (SLB) as previously described [6]. A possible UIP pattern on HRCT was recorded as UIP, as possible UIP on HRCT has been shown to strongly correlate with histopathologic UIP [26, 27]. SLB pattern was considered the final radiographic/histopathologic pattern when there was discordance between the two diagnostic modalities. All but two patients with an HRCT pattern inconsistent with UIP had UIP on SLB. The two patients without UIP on SLB were considered to have IPF given a strong family history of pulmonary fibrosis. Vital status was determined using review of medical records, telephone communication and the social security death index. Follow-up time was censored on January 1, 2016. Patients were excluded if they refused consent, did not undergo ANA testing, had only one ILD clinic visit or did not have UIP by HRCT or SLB. Genotype data was available for a subset of individuals included in this study, which composed the replication cohort of our recent study demonstrating that NAC response may vary by *TOLLIP* rs3750920 genotype [12]. Methods for genotype determination were previously reported [12].

### Statistical analysis

Continuous variables were reported as means with standard deviation (SD) or medians with interquartile range (IQR), as appropriate. Categorical variables were reported as counts and percentages. Categorical data were compared using the Chi-square test or Fisher’s exact test, as appropriate. Survival was assessed using unadjusted log rank testing along with univariate and multivariable Cox proportional hazards regression after ensuring the proportional hazards assumption was met for each model. Survival curves are plotted using the Kaplan-Meier survival estimator. Survival time was defined as time from diagnostic test (SLB or HRCT) to death, transplant, or censoring date (January 1, 2016). The gender, age, physiology (GAP) index was used as a surrogate for disease severity in outcomes modeling, as this index accounts for individuals unable to perform the DLCO maneuver and has been shown to be a reliable predictor of mortality [28]. Given the limited sample size for each of the immunosuppressant and anti-fibrotic therapies, therapeutic classes were used for outcomes modeling. Statistical significance was considered at *p* < 0.05. All statistical analyses were performed using Stata (StataCorp. 2013. Release 13. College Station, TX).

## Results

Of 293 patients meeting inclusion criteria (Table 1), 216 (73.7) had IPF and 77 (26.3%) had IPAF-UIP. Among those with IPAF-UIP, 31 (40.2%) carried a previous diagnosis of IPF before application of IPAF criteria. The mean age of the entire UIP cohort was 67.7 (±8.8) years with a male predominance (68.6%). The predominant race was white (82.3%), followed by African American (8.2%), Hispanic (6.1%) and Asian (3.4%). A history of smoking was observed in 68% of the cohort. A positive autoantibody was observed in 167 (61.6%) individuals, 148 (50.5%) of which had a positive ANA (50.5%). On HRCT, UIP was the predominant pattern (67.5%) followed by possible UIP (17.1%) and inconsistent with UIP (15.4%). All patients with possible UIP on HRCT who underwent SLB had histologic UIP (*n* = 28). The mean percent predicted FVC and DLCO was 66.5% and 50.3%, respectively. The mean GAP score was 4 (±1.6).

**Table 1:** Baseline Characteristics and Outcomes

Variable	IPF Cohort (*n* = 216)	IPAF-UIP Cohort (*n* = 77)	Combined UIP Cohort (*n* = 293)^a^
Age, mean (±SD)	69.3 (7.8)	63.3 (9.9)	67.7 (8.8)
Male Gender, n (%)	160 (74.1)	41 (53.3)	201 (68.6)
Race, n (%)			
White	182 (84.3)	59 (76.6)	241 (82.3)
African American	15 (6.9)	9 (11.7)	24 (8.2)
Hispanic	10 (4.6)	8 (10.4)	18 (6.1)
Asian	9 (4.2)	1 (1.3)	10 (3.4)
Any auto-antibody (+)	93 (43.1)	74 (96.1)	167 (57.0)
ANA (+) ^b^	84 (38.9)	64 (83.1)	148 (50.5)
HRCT, n (%)			
UIP	156 (72.2)	41 (54)	197 (67.5)
Possible UIP	36 (16.7)	14 (18.4)	50 (17.1)
Inconsistent with UIP	24 (11.1)	21 (27.6)	45 (15.4)
UIP by SLB ^c^, n (%)	93 (97.9)	55 (100)	148 (98.7)
FVC (% pred), mean (±SD)	67.5 (17.4)	63.5 (16.9)	66.5 (17.4)
DLCO (% pred), mean (±SD)	51.1 (17.1)	48.1 (16.8)	50.3 (17.1)
GAP Score, mean (±SD)	4.1 (1.6)	3.7 (1.6)	4.0 (1.6)
Outcomes			
Death	108 (50)	35 (45.5)	143 (48.8)
Transplant	14 (6.5)	14 (18.2)	28 (9.6)

*Abbreviations*: *IPF* idiopathic pulmonary fibrosis, *IPAF* interstitial pneumonia with autoimmune features, *ANA* anti-nuclear antibody, *HRCT* high-resolution computed tomography, *UIP* usual interstitial pneumonia, *SLB* surgical lung biopsy, *FVC* forced vital capacity, *DLCO* diffusion capacity of the lung for carbon monoxide, *GAP* gender, age, physiology

^a^Exception for n: Other auto-antibody (*n* = 88); HRCT (*n* = 292); UIP by SLB (*n* = 150); DLCO (*n* = 272)

^b^ANA titer ≥1:320 or nucleolar or centromere staining pattern at any titer

^c^Two patients without UIP by SLB were deemed to have IPF due to family history of pulmonary fibrosis

Fifty-eight individuals were exposed to NAC, while 235 were not (Table 2). Of these patients, 29 (50%) received NAC monotherapy, while the rest received NAC and an immunosuppressant (*n* = 15) or anti-fibrotic (*n* = 11). Three patients received NAC along with immunosuppressant and anti-fibrotic during the study period. The median NAC exposure time among individuals treated at our institution was 9.8 months [IQR 4.1–18.1]. There was no significant difference in prednisone, mycophenolate mofetil, pirfenidone or nintedanib exposure use between NAC exposed and non-exposed cohorts. There was significantly more azathioprine exposure in the NAC exposed group when compared to the NAC non-exposed group (13.8% vs. 3.8%, respectively; *p* = 0.004) but a similar overall exposure to immunosuppression between groups (25.9% vs 22.8%, *p* = 0.64). Forty-nine percent of patients died during the follow-up period and nearly 10% received a lung transplant.

**Table 2:** UIP Cohort Treatment Exposure During Study Period

Therapy	NAC Exposed (*n* = 58)	NAC Unexposed (*n* = 235)	*p*-value
NAC monotherapy	29 (50)	0 (0)	< 0.001
Immunosuppressant exposure^a^	15 (25.9)	54 (22.8)	0.64
Prednisone	15 (25.9)	53 (22.6)	0.59
Azathioprine	8 (13.8)	9 (3.8)	0.004
Mycophenolate mofetil	3 (6.0)	3 (2.8)	0.38
Anti-fibrotic exposure	11 (18.9)	29 (12.3)	0.19
Pirfenidone	10 (17.2)	24 (10.2)	0.13
Nintedanib	1 (1.8)	6 (2.6)	1

*Abbreviations*: *UIP* usual interstitial pneumonia, *NAC* N-acetylcysteine

^a^Azathioprine/prednisone or mycophenolate/prednisone

When assessing relevant variables for association with transplant-free survival (TFS) (Table 3), anti-fibrotic exposure was associated with improved TFS (HR 0.28; 95% CI 0.14–0.57; *p* < 0.001), while ANA seropositivity (HR 1.46, 95% CI 1.07–1.98; *p* = 0.02) and each increase in GAP score (HR 1.36, 95% CI 1.23–1.51; *p* < 0.001) were associated with worse TFS in unadjusted analysis. Diagnosis (IPAF vs. IPF), NAC exposure, prednisone exposure and azathioprine exposure were not associated with differential TFS risk. These survival associations remained in a multivariable model adjusted for ANA status, diagnosis, NAC exposure, immunosuppressant exposure, anti-fibrotic exposure and GAP score.

**Table 3:** Variables Predicting Survival in UIP Cohort

	Unadjusted (*n* = 293)			Adjusted (*n* = 293)		
Characteristic	HR	*p*-value	95% CI	HR	*p*-value	95% CI
ANA (+) ^a^	1.46	0.02	1.07–1.98	1.59	0.007	1.13–2.23
NAC exposure	0.74	0.13	0.50–1.09	0.68	0.05	0.44–1.0
IPAF diagnosis ^b^	1.11	0.52	0.80–1.57	0.99	0.97	0.67–1.48
Immunosuppressant exposure ^c^	1.05	0.77	0.74–1.49	0.94	0.75	0.64–1.38
Anti-fibrotic exposure ^d^	0.28	< 0.001	0.14-0.57	0.35	0.004	0.17–0.72
GAP Score	1.36	< 0.001	1.23–1.51	1.36	< 0.001	1.23–1.51

*Abbreviations*: *IPF* idiopathic pulmonary fibrosis, *IPAF* interstitial pneumonia with autoimmune features, *UIP* usual interstitial pneumonia, *NAC* N-acetylcysteine, *ANA* antinuclear antibody, *GAP* gender, age, physiology

^a^ANA titer ≥1:320 or nucleolar or centromere staining pattern at any titer

^b^Compared to IPF diagnosis

^c^Prednisone, azathioprine or mycophenolate

^d^Pirfenidone or nintedanib

**Fig. 1:**
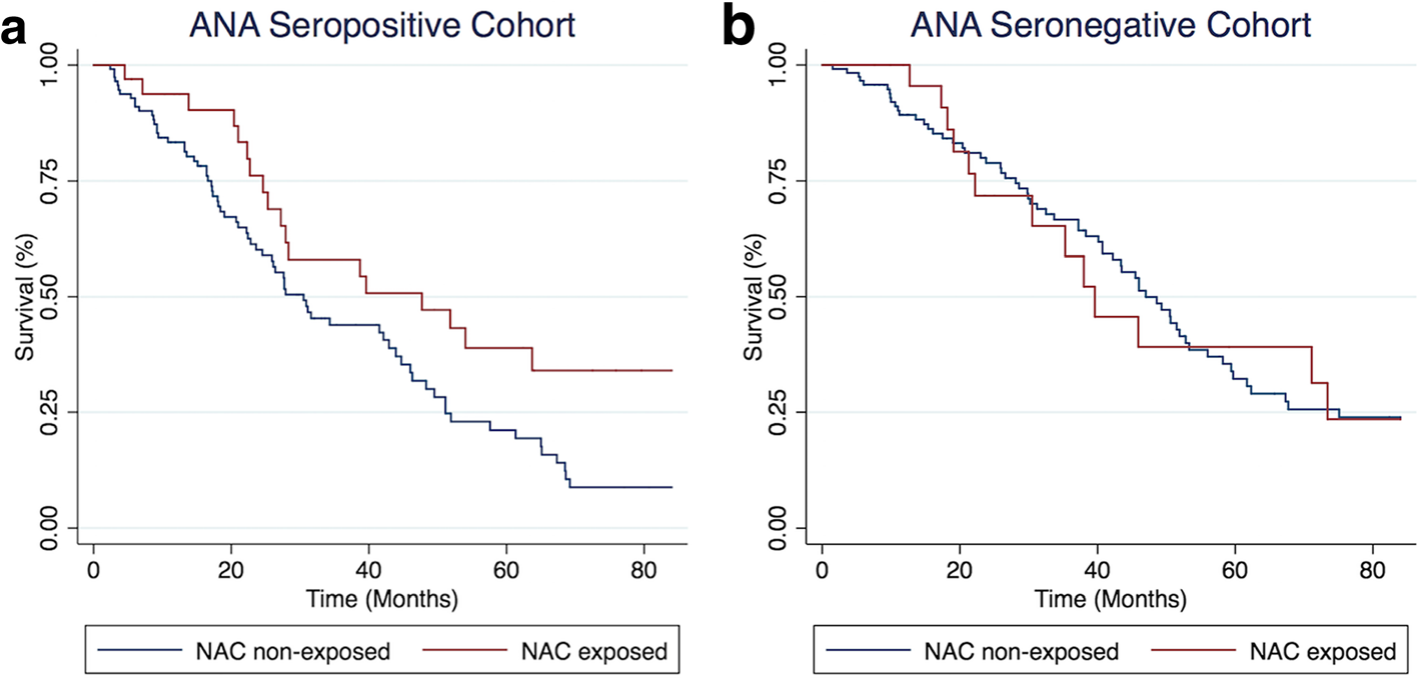
Transplant-free survival among NAC exposed and non-exposed individuals with UIP stratified by ANA antibody status. Among ANA seropositive individuals (**a**), NAC exposed individuals demonstrated improved survival compared to NAC non-exposed individuals (p_logrank_ = 0.02; HR 0.55, 95% CI 0.32–0.92). Among ANA seronegative individuals (**b**), survival did not differ between NAC exposed and non-exposed individuals (p_logrank_ *p* = 0.92; HR 1.03, 95% CI 0.56–1.87)

After stratification of the cohort by ANA seropositivity (Fig. 1) (Table 4), NAC was associated with significantly improved TFS among ANA seropositive patients (p_logrank_ = 0.02; HR 0.55, 95% CI 0.32–0.92), but not among ANA seronegative patients (p_logrank_ *p* = 0.92; HR 1.03, 95% CI 0.56–1.87). After adjustment for diagnosis (IPAF vs. IPF), immunosuppressant exposure, anti-fibrotic exposure and GAP score, the association between NAC exposure and improved TFS among ANA seropositive patients was maintained (HR 0.51, 95% CI 0.30–0.87; *p* = 0.01). Anti-fibrotic exposure was associated with improved TFS in both groups, but only reached statistical significance in the ANA seronegative group (HR 0.16, 95% CI 0.05–0.51; *p* = 0.002). IPAF diagnosis was also associated with improved TFS in the ANA seronegative group after multivariable adjustment (HR 0.37; 95% CI 0.14–0.99; *p* = 0.05).

**Table 4:** Multivariable-adjusted NAC-associated mortality risk stratified by ANA seropositivity^a^

	ANA (+) ^a^ (*n* = 148)				ANA (−) (*n* = 145)			
Characteristic	n	HR	*p*-value	95% CI	n	HR	*p*-value	95% CI
NAC exposure	33	0.51	0.01	0.30–0.87	25	1.26	0.45	0.69–2.32
IPAF diagnosis ^b^	64	1.23	0.37	0.78–1.95	13	0.37	0.05	0.14–0.99
Immunosuppressant exposure ^c^	35	0.85	0.54	0.50–1.43	34	1.32	0.32	0.77–2.28
Anti-fibrotic exposure ^d^	20	0.67	0.4	0.27–1.70	22	0.16	0.002	0.05–0.51
GAP Score	148	1.41	< 0.001	1.23–1.61	144	1.27	0.005	1.08–1.50

*Abbreviations*: *NAC* N-acetylcysteine, *ANA* anti-nuclear antibody, *IPAF* interstitial pneumonia with autoimmune features, *GAP* gender, age, physiology

^a^ANA titer ≥1:320 or nucleolar or centromere staining pattern at any titer

^b^Compared to IPF diagnosis

^c^Prednisone, azathioprine or mycophenolate

^d^Pirfenidone or nintedanib

Significant interaction between NAC exposure and ANA seropositivity (p_interaction_ = 0.04) was observed in a multivariable model that included NAC*ANA interaction term and diagnosis, immunosuppressant exposure, anti-fibrotic exposure and GAP score as covariates. No significant interaction was observed between NAC exposure and diagnosis, immunosuppressant exposure or anti-fibrotic exposure using similar models. Sensitivity analysis was performed to explore the association between NAC exposure and TFS at various ANA titers (Table 5). NAC therapy was associated with improved TFS at all titers, but reached statistical significance at titers ≥1:320. The strongest effect size was observed in those with an ANA titer ≥1:1280, but there were a small number of individuals (*n* = 14) in this group.

**Table 5:** NAC exposure TFS Risk^a^ at increasing ANA titers

ANA Titer	n	HR	*p*-value	95% CI
≥1:160	42	0.67	0.08	0.42–1.05
≥1:320	33	0.51	0.01	0.30–0.87
≥1:640	23	0.52	0.05	0.28–0.99
≥1:1280	14	0.4	0.04	0.16–0.97

*Abbreviations*: *NAC* N-acetylcysteine, *TFS* transplant-free survival, *ANA* anti-nuclear antibody

^a^Adjusted for diagnosis, immunosuppressant exposure, anti-fibrotic exposure and GAP score

Subgroup analysis was performed to further explore the association between NAC and improved TFS among those with ANA seropositivity. NAC remained associated with improved TFS in those with ANA seropositivity when excluding individuals who received immunosuppression (HR 0.41, 95% CI 0.21–0.81; *p* = 0.01) (Additional file 1: Table S1), when excluding individuals who received an anti-fibrotic (HR 0.57; 95% CI 0.33–0.99; *p* = 0.05) (Additional file 2: Table S2) and when excluding individuals who received either an immunsuppressant or anti-fibrotic (HR 0.46; 95% CI 0.23–0.93; *p* = 0.03) (Additional file 3: Table S3). A similar effect size was observed when considering only those with a diagnosis of IPF (HR 0.43, 95% CI 0.21–0.85; *p* = 0.02) (Additional file 4: Table S4) and there were an insufficient number of observations to conduct this analysis in an IPAF-only cohort.

We then assessed whether ANA status was associated with rs3750920 (*TOLLIP*) genotype (Table 6). Among those for whom these data were available (*n* = 116), there was a significant difference in genotype frequency between ANA seropositive and seronegative patients (*p* = 0.03). This difference was driven primarily by differences in the CT and TT genotypes between groups. When considering only those with a TT genotype, a higher percentage of ANA seropositive compared to seronegative patients carried this genotype (40.4% vs. 25.0%), but this difference was of marginal statistical significance (*p* = 0.08) in head-to-head comparison. These findings persisted after stratification of the cohort by NAC exposure (Additional file 5: Table S5), but the analysis was limited by sample sizes in the NAC exposed group.

**Table 6:** rs3750920 (*TOLLIP*) genotypes stratified by ANA status

ANA Status	CC	CT	TT	*p*-value
ANA (−) (*n* = 64)	9 (14.1)	39 (60.9)	16 (25)	0.03
ANA (+) (*n* = 52)	12 (23.1)	19 (36.5)	21 (40.4)	

## Discussion

In this investigation, we showed that NAC responsiveness varies by the presence of circulating ANA. We found that NAC exposure was associated with improved TFS among ANA seropositive individuals with UIP, but not among those who were seronegative. This association appeared to strengthen as ANA titer increased. We also showed that there may be an increased frequency of the rs3750920 (*TOLLIP*) TT genotype in ANA seropositive patients, which may reflect why such patients had improved survival when exposed to NAC therapy. This study, to our knowledge, is the first to demonstrate differential UIP treatment response based on the presence of circulating autoantibodies. These findings support our previous work showing that NAC may benefit a subset of patients with IPF [12].

The mechanism by which NAC responsiveness varies in the presence of circulating autoantibodies remains unclear, but TLR signaling may play a role. The TLRs are critical mediators of innate immunity and TLR2, 3, 4, and 7 are particularly important in airway host defense through their recognition of inhaled and circulating microbial antigens. Activation of TLRs generally occurs through recognition of exogenous pathogen-associated molecular patterns, which triggers inflammatory signaling pathways that facilitate infection eradication and stimulate wound healing [29]. *TOLLIP* encodes the toll-interacting protein, an inhibitory adaptor protein that acts downstream from TLRs.

TLR activation can also occur by recognition of endogenous damage-associated molecular patterns (DAMPs), which arise from molecular fragments released from damaged host cells [30]. Nucleic acid DAMPs within self-antigens bound to autoantibodies have been shown to activate TLR3 and TLR7 in systemic lupus erythematosis [16–19, 31]. The high mobility group box protein 1 DAMP, released by damaged synovial cells in patients with rheumatoid arthritis has been shown to activate TLR4 [32]. Extracellular matrix DAMPs that arise during dermal wound healing in patients with scleroderma have also been shown to activate TLR4 and enhance the sensitivity of skin fibroblasts to the fibrogenic effect of transforming growth factor-β1F [33].

Activation of TLR2, 3, 4 and 7 initiates oxidative signaling, leading to an environment of increased oxidative stress [22, 24, 34, 35]. NAC has been shown to blunt this oxidative stress response and can do so in a dose-dependent manner [22–24]. These observations suggest that NAC may be mitigating harmful oxidative stress caused by TLR activation by self-antigen/autoantibody complexes in ANA seropositive patients with UIP. Conversely, we also observed that anti-fibrotic therapy may be more efficacious in patients without circulating ANA (Table 4). The reason for this remains unclear, but may indicate that the blockade of fibrotic pathways is more effective in the absence of immune activation.

In addition to our primary findings, we also found that ANA seropositivity was associated with worse outcomes in this cohort (Table 3). This runs counter to that observed by others, [20, 36] including an earlier study of autoimmune-featured ILD by our group, [37] which found that circulating autoantibodies either predicted improved outcomes, or had no impact on survival. The percentage of ANA seropositive patients in our cohort is higher than that reported in other cohorts around the world, [20, 36] which may explain the discordant results with regard to mortality risk, and highlights the high likelihood of regional variability in autoantibody seropositivity. We also found that a subset of patients with IPAF, namely those in the ANA seronegative group, had improved TFS compared those with IPF (Table 4). This finding supports our earlier work that identified individuals with IPAF with a favorable prognosis compared to IPF despite a similar background of UIP [6]. These individuals tended to have physical manifestations of CTD (IPAF clinical domain) along with features of CTD on HRCT, including non-specific interstitial pneumonia and/or organizing pneumonia (IPAF morphologic domain). Given the variability in presentation and outcomes of recently characterized IPAF cohorts, [5, 6, 38] more research is needed to identify the optimal therapy for those meeting IPAF criteria.

Our study has several limitations. First the retrospective nature of this investigation did not allow for assignment of causation, only association. It is possible that the presence of unmeasured systematic differences between NAC exposed and unexposed individuals influenced our results. Next, our cohort was derived from a single center, which limits generalizability, especially since the prevalence of ANA seropositivity appears to vary substantially by geographical region, [20, 36, 37] and our center has a higher autoantibody frequency than most others. Complete serologic data was not available for all patients, as current recommendations do not advise comprehensive serologic testing for all patients [25]. Our study was also limited by its small sample size, especially in the NAC exposed group, which precluded multi-level modeling of NAC responsiveness at various ANA titers and within IPF and IPAF disease states. The imbalance in immunosuppressant and anti-fibrotic therapies between NAC exposed and non-exposed cohorts also potentially introduced bias, as did the variability in exposure time with regard to NAC, immunosuppressants and anti-fibrotics. We conducted a sensitivity analysis to explore this limitation and found consistent results after exclusion of patients exposed to an immunosuppressant and/or anti-fibrotic therapy. (Additional file 1: Table S1, Additional file 2: Table S2, Additional file 3: Table S3). Next, our reliance on patient-reported NAC use did not allow us to confirm NAC duration or dosage for a large minority of patients exposed to NAC prior to referral to our institution. As a result, we relied on categorical NAC exposure for our modeling. The median duration of NAC exposure among patients treated at our institution was 9 months, suggesting a prolonged course may be necessary.

## Conclusion

From this study we conclude that antioxidant therapy may be efficacious in a subset of patients with UIP, specifically those with circulating ANA. These findings should be viewed as hypothesis generating and support the collection of ANA data in the design of any future NAC clinical trials to explore further these findings. Replication of these findings would advance the era of precision medicine through identification of an immunologically predisposed subset of patients with UIP for whom NAC may be an efficacious adjunct to anti-fibrotic therapy.

## Additional files

Additional file 1: Table S1. Multivariable-adjusted NAC-associated mortality risk stratified by ANA seropositivity after exclusion of patients receiving immunosuppression. (DOCX 63 kb)

Additional file 2: Table S2. Multivariable-adjusted NAC-associated mortality risk stratified by ANA seropositivity after exclusion of patients receiving an anti-fibrotic. (DOCX 61 kb)

Additional file 3: Table S3. Multivariable-adjusted NAC-associated mortality risk stratified by ANA seropositivity after exclusion of patients receiving immunosuppression or an anti-fibrotic. (DOCX 56 kb)

Additional file 4: Table S4. Multivariable-adjusted NAC-associated mortality risk stratified by ANA seropositivity in only those with IPF. (DOCX 58 kb)

Additional file 5: Table S5. rs3750920 (*TOLLIP*) genotypes stratified by NAC Exposure and ANA Status. (DOCX 42 kb)

## Abbreviations

ANA: Antinuclear antibody
ATS: American Thoracic Society
CI: Confidence interval
CTD: Connective tissue disease
DAMP: Damage-associated molecular pattern
DLCO: Diffusion capacity of the lung for carbon monoxide
ERS: European Respiratory Society
FVC: Forced vital capacity
GAP: Gender, age, physiology
HR: Hazard ratio
HRCT: High-resolution computed tomagraphy
IIP: Idiopathic interstitial pneumonia
ILD: Interstitial lung disease
IPAF: Interstitial pneumonia with autoimmune features
IPF: Idiopathic pulmonary fibrosis
IQR: Interquartile range
IRB: Institutional review board
NAC: *N*-acetylcysteine
PANTHER: Prednisone, azathioprine and N-acetylcysteine for pulmonary fibrosis trial
SD: Standard deviation
SLB: Surgical lung biopsy
TFS: Transplant-free survival
TLR: Toll-like receptor
UIP: Usual interstitial pneumonia
