# Supplementary material for: N-acetylcysteine exposure is associated with improved survival in anti-nuclear antibody seropositive patients with usual interstitial pneumonia
Source: BMC Pulm Med. 2018 Feb 8;18:30. doi: 10.1186/s12890-018-0599-3 (PMC5806226; doi:10.1186/s12890-018-0599-3)
Supplement: Supplementary file 1 — Multivariable-adjusted NAC-associated mortality risk stratified by ANA seropositivity after exclusion of patients receiving immunosuppression. (DOCX 63 kb) [file 12890_2018_599_MOESM1_ESM.docx]

| **Table E1. Multivariable-adjusted NAC-associated mortality risk stratified by ANA seropositivity after exclusion of patients receiving immunosuppression** | | | | | | | |
| --- | --- | --- | --- | --- | --- | --- | --- |
|  | **ANA (+)* (n=113)** | | |  | **ANA (-) (n=110)** | | |
| **Characteristic** | **HR** | **p-value** | **95% CI** |  | **HR** | **p-value** | **95% CI** |
| NAC exposure | 0.41 | **0.01** | 0.21-0.81 |  | 0.83 | 0.64 | 0.38-1.81 |
| IPAF diagnosis** | 1.19 | 0.5 | 0.71-2.00 |  | 1.54 | 0.57 | 0.34-6.89 |
| Anti-fibrotic exposure*** | 0.63 | 0.39 | 0.22-1.80 |  | 0.22 | **0.01** | 0.07-0.73 |
| GAP Score | 1.41 | **<0.001** | 1.21-1.65 |  | 1.25 | **0.05** | 1.0-1.56 |
| Abbreviations: NAC=N-acetylcysteine; ANA=anti-nuclear antibody; IPAF=interstitial pneumonia with autoimmune features; GAP=gender, age, physiology | | | | | | | |
| * ANA titer ≥ 1:320 or nucleolar or centromere staining pattern at any titer | | | | | | | |
| ** Compared to IPF diagnosis | | | | | | | |
| *** Pirfenidone or nintedanib |  |  |  |  |  |  |  |
