# Supplementary material for: N-acetylcysteine exposure is associated with improved survival in anti-nuclear antibody seropositive patients with usual interstitial pneumonia
Source: BMC Pulm Med. 2018 Feb 8;18:30. doi: 10.1186/s12890-018-0599-3 (PMC5806226; doi:10.1186/s12890-018-0599-3)
Supplement: Supplementary file 2 — Multivariable-adjusted NAC-associated mortality risk stratified by ANA seropositivity after exclusion of patients receiving an anti-fibrotic. (DOCX 61 kb) [file 12890_2018_599_MOESM2_ESM.docx]

| **Table E2. Multivariable-adjusted NAC-associated mortality risk stratified by ANA seropositivity after exclusion of patients receiving an anti-fibrotic** | | | | | | | |
| --- | --- | --- | --- | --- | --- | --- | --- |
|  | **ANA (+)* (n=128)** | | |  | **ANA (-) (n=122)** | | |
| **Characteristic** | **HR** | **p-value** | **95% CI** |  | **HR** | **p-value** | **95% CI** |
| NAC exposure | 0.57 | **0.05** | 0.33-0.99 |  | 1.39 | 0.29 | 0.76-2.55 |
| IPAF diagnosis** | 1.45 | 0.13 | 0.90-2.33 |  | 0.37 | **0.05** | 0.14-0.98 |
| Immunosuppressant exposure*** | 0.74 | 0.27 | 0.43-1.27 |  | 1.35 | 0.29 | 0.78-2.34 |
| GAP Score | 1.37 | **<0.001** | 1.20-1.57 |  | 1.31 | **0.002** | 1.10-1.55 |
| Abbreviations: NAC=N-acetylcysteine; ANA=anti-nuclear antibody; IPAF=interstitial pneumonia with autoimmune features; GAP=gender, age, physiology | | | | | | | |
| * ANA titer ≥ 1:320 or nucleolar or centromere staining pattern at any titer | | | | | | | |
| ** Compared to IPF diagnosis | | | | | | | |
| *** Prednisone, azathioprine or mycophenolate | | |  |  |  |  |  |
