# Supplementary material for: N-acetylcysteine exposure is associated with improved survival in anti-nuclear antibody seropositive patients with usual interstitial pneumonia
Source: BMC Pulm Med. 2018 Feb 8;18:30. doi: 10.1186/s12890-018-0599-3 (PMC5806226; doi:10.1186/s12890-018-0599-3)
Supplement: Supplementary file 3 — Multivariable-adjusted NAC-associated mortality risk stratified by ANA seropositivity after exclusion of patients receiving immunosuppression or an anti-fibrotic. (DOCX 56 kb) [file 12890_2018_599_MOESM3_ESM.docx]

| **Table E3. Multivariable-adjusted NAC-associated mortality risk stratified by ANA seropositivity after exclusion of patients receiving immunosuppression or an anti-fibrotic** | | | | | | | | | |
| --- | --- | --- | --- | --- | --- | --- | --- | --- | --- |
|  | **ANA (+)* (n=94)** | | | |  | | **ANA (-) (n=90)** | | |
| **Characteristic** | **HR** | **p-value** | **95% CI** |  | | **HR** | | **p-value** | **95% CI** |
| NAC exposure | 0.46 | **0.03** | 0.23-0.93 |  | | 0.97 | | 0.94 | 0.45-2.11 |
| IPAF diagnosis** | 1.41 | 0.2 | 0.83-2.41 |  | | 1.39 | | 0.67 | 0.31-6.26 |
| GAP Score | 1.38 | **<0.001** | 1.17-1.61 |  | | 1.32 | | **0.02** | 1.05-1.67 |
| Abbreviations: NAC=N-acetylcysteine; ANA=anti-nuclear antibody; IPAF=interstitial pneumonia with autoimmune features; GAP=gender, age, physiology | | | | | | | | | |
| * ANA titer ≥ 1:320 or nucleolar or centromere staining pattern at any titer | | | | | | | | | |
| ** Compared to IPF diagnosis | | | | | | | | | |
