# Supplementary material for: N-acetylcysteine exposure is associated with improved survival in anti-nuclear antibody seropositive patients with usual interstitial pneumonia
Source: BMC Pulm Med. 2018 Feb 8;18:30. doi: 10.1186/s12890-018-0599-3 (PMC5806226; doi:10.1186/s12890-018-0599-3)
Supplement: Supplementary file 4 — Multivariable-adjusted NAC-associated mortality risk stratified by ANA seropositivity in only those with IPF. (DOCX 58 kb) [file 12890_2018_599_MOESM4_ESM.docx]

| **Table E4. Multivariable-adjusted NAC-associated mortality risk stratified by ANA seropositivity in only those with IPF** | | | | | | | |
| --- | --- | --- | --- | --- | --- | --- | --- |
|  | **ANA (+)* (n=84)** | | |  | **ANA (-) (n=131)** | | |
| **Characteristic** | **HR** | **p-value** | **95% CI** |  | **HR** | **p-value** | **95% CI** |
| NAC exposure | 0.43 | **0.02** | 0.21-0.85 |  | 1.1 | 0.77 | 0.57-2.11 |
| Immunosuppressant exposure** | 0.86 | 0.76 | 0.34-2.20 |  | 1.5 | 0.14 | 0.87-2.59 |
| Anti-fibrotic exposure*** | 1.36 | 0.53 | 0.52-3.57 |  | 0.15 | **0.002** | 0.05-0.49 |
| GAP Score | 1.54 | **<0.001** | 1.28-1.86 |  | 1.21 | **0.02** | 1.01-1.43 |
| Abbreviations: NAC=N-acetylcysteine; ANA=anti-nuclear antibody; GAP=gender, age, physiology | | | | | | | |
| * ANA titer ≥ 1:320 or nucleolar or centromere staining pattern at any titer | | | | | | | |
| ** Prednisone, azathioprine or mycophenolate | | |  |  |  |  |  |
| *** Pirfenidone or nintedanib |  |  |  |  |  |  |  |
