# Supplementary material for: N-acetylcysteine exposure is associated with improved survival in anti-nuclear antibody seropositive patients with usual interstitial pneumonia
Source: BMC Pulm Med. 2018 Feb 8;18:30. doi: 10.1186/s12890-018-0599-3 (PMC5806226; doi:10.1186/s12890-018-0599-3)
Supplement: Supplementary file 5 — rs3750920 (TOLLIP) genotypes stratified by NAC Exposure and ANA Status. (DOCX 42 kb) [file 12890_2018_599_MOESM5_ESM.docx]

| **Table E5. rs3750920 (*TOLLIP*) genotypes stratified by NAC Exposure and ANA Status** | | | | | | | | |
| --- | --- | --- | --- | --- | --- | --- | --- | --- |
| **ANA Status** | **NAC exposed (n=20)** | | | | **NAC non-exposed (n=96)** | | | |
|  | **CC** | **CT** | **TT** | **p-value** | **GG** | **GT** | **TT** | **p-value** |
| **ANA (-) (n=64)** | 2 (20) | 7 (70) | 1 (10) | 0.16 | 7 (12.9) | 32 (59.3) | 15 (27.8) | 0.02 |
| **ANA (+) (n=52)** | 0 (0) | 6 (60) | 4 (40) |  | 12 (28.6) | 13 (30.9) | 17 (40.5) |  |
